# Validation of the competitive attention test: behavioral reliability and construct-relevant associations across the lifespan

**DOI:** 10.3389/fpsyg.2026.1720923

**Published:** 2026-05-21

**Authors:** Nicola Thibault, Aurélie Grandjean, Marie-Frédérique Bernier, Émilie Cloutier-Debaque, Steven Laureys, Aurélie Bidet-Caulet, Roxane S. Hoyer

**Affiliations:** 1 CERVO Brain Research Centre, Québec, QC, Canada; 2 School of Psychology, Laval University, Québec, QC, Canada; 3 Faculty of Medicine, Laval University, Québec, QC, Canada; 4 Joint International Research Unit on Neuroplasticity, Laval University, Québec, QC, Canada; 5 Aix Marseille Univ, Inserm, INS, Inst Neurosci Syst, Marseille, France; 6 GIGA Consciousness Research Unit and Coma Science Group, Liège University, Liège, Belgium; 7 International Consciousness Science Institute, Hangzhou Normal University, Hangzhou, China; 8 Université Claude Bernard Lyon 1, INSERM, CNRS, Centre de Recherche en Neurosciences de Lyon, Bron, France

**Keywords:** attention, distractibility, neuropsychology, testing, validation

## Abstract

**Introduction:**

The Competitive Attention Test (CAT) assesses distractibility as a multidimensional construct derived from cognitive neuroscience models of attention, capturing voluntary and involuntary attentional processes and their underlying mechanisms using ecologically relevant stimuli. To validate its psychometric properties, we conducted two experiments examining construct-relevant reliability and validity evidence in a lifespan sample (ages 6–89).

**Methods:**

Experiment 1 assessed internal consistency (*N =* 520) and inter-rater reliability (*N =* 114) of indices related to speed, variability, phasic arousal, and accuracy. Experiment 2 evaluated short-term test–retest reliability (*N =* 79), convergent validity with established attention measures (TAP-M, KiTAP; *n =* 38), and divergent validity with respect to broader cognitive abilities assessed using Wechsler intelligence scales.

**Results:**

Experiment 1 confirmed coherent clustering and examiner stability of the CAT indices. In Experiment 2, most indices showed temporal stability, meaningful associations with attention-specific constructs, and minimal overlap with reasoning abilities.

**Conclusion:**

These findings support the CAT as a reliable, theoretically informed measure of attentional control, with an ecologically valid design suited to research and potential clinical assessments of distractibility from 6 to 89-year-old.

## 1 Introduction

Attention is not a unitary ability, nor is it stable across the lifespan. From childhood through older age, goal-directed focus, susceptibility to distraction, and the inhibition of premature responses show substantial change linked to cerebral development (Blakemore and Choudhury, 2006, Toga et al., 2006). Lifespan studies further suggest that multiple components, including sustained attention, phasic arousal facilitation, stimulus-driven capture, and motor impulsivity, follow nonlinear and partially dissociable trajectories across the lifespan, with gains from childhood to young adulthood and partial decline later in life (Hoyer et al., 2023a, b). In this context, characterizing normative maturation and aging cannot rely on unitary measures of attention, but instead requires paradigms that can jointly and selectively quantify voluntary orienting, stimulus-driven capture, and impulsive responding.

Attention is a cornerstone of neuropsychological assessment; survey data indicate that it is the most frequently evaluated cognitive domain by clinical neuropsychologists (Rabin et al., 2005). However, “attention” is often treated as a single construct despite encompassing multiple dissociable processes, including maintaining focus on relevant stimuli (sustained attention; Langner and Eickhoff, 2013; Parasuraman et al., 1989), selecting relevant information while filtering competing inputs (selective attention; Desimone and Duncan, 1995; Oberauer, 2019; Plebanek and Sloutsky, 2019), and orienting attention toward task-relevant information (voluntary orienting) or reorienting following distraction by salient events (Petersen and Posner, 2012; Posner, 1980; Wetzel and Schröger, 2014). Within this framework, distractibility refers to the propensity of task-irrelevant or unexpected events to capture attention during ongoing activity, whereas distraction denotes the measurable performance cost that follows from such capture (Bidet-Caulet et al., 2015; Wetzel and Schröger, 2014). Salient events can be adaptive by transiently boosting alertness and prioritizing potentially important information (e.g., threat; Max et al., 2015; Yerkes and Dodson, 1908). However, excessive distractibility disrupts academic, occupational, and social functioning, and is a prominent feature of several clinical conditions, including attention deficit disorder with/without Hyperactivity (ADHD; Barkley, 1997; Sonuga-Barke and Castellanos, 2007).

Clinically, increased distraction characterizes various neuropsychological disorders. Patients with ADHD frequently report being persistently diverted by irrelevant stimuli (Barkley, 1997). Similar vulnerabilities have been reported in individuals with traumatic brain injuries or neurodegenerative diseases (McDonald et al., 2002; Perry and Hodges, 1999). However, existing assessment tools, such as the Conners CPT 3 and TAP/KiTAP batteries, primarily index sustained attention or executive load but rarely include irrelevant distractors (Conners et al., 2018; Leclercq and Zimmermann, 2004). Consequently, clinicians face challenges in determining whether functional impairment arises from involuntary attentional capture, impulsivity, or lapses in sustained attention. A brief, lifespan-appropriate test incorporating ecological distractors would thus enable finer-grained attention profiling, and bridge behavioral assessment (Hoyer et al., 2021, 2023a, b) with neuroimaging research (ElShafei et al., 2018, Bidet-Caulet et al., 2015, Grandjean et al., 2024). Historically, most neuropsychological tests of attention were developed within a behavioral framework, with constructs defined at the performance level before their neural mechanisms were identified. Although this approach has produced robust clinical tools, it has also promoted broad attentional constructs that do not always align with dissociations observed at the neural level (Sherman et al., 2023).

To address these limitations, the Competitive Attention Test (CAT) was developed as a single, neuroscience-informed protocol assessing multiple facets of distractibility, grounded in models that distinguish voluntary orienting, stimulus-driven capture, arousal modulation, and motor inhibition as partially independent processes, with task parameters designed to reflect these distinctions directly from neural mechanisms rather than inferring them *post-hoc* from behavioral performance (Bidet-Caulet et al., 2015; Hoyer et al., 2021, 2023). During the CAT, participants detect auditory targets preceded by unexpected distractors, generating a range of behavioral indices including reaction time, omission errors, and various forms of false alarms. By integrating ecologically relevant distractors (e.g., ringtones, alarms), the CAT extends traditional sustained attention tasks by probing situations in which attention can be involuntarily captured or facilitated by salient events. Crucially, the CAT also manipulates voluntary attention through cues predicting target location (Bidet-Caulet et al., 2015), allowing assessment of how individuals allocate attentional resources depending on whether the target side is predictable or not (Grandjean et al., 2024; Petersen and Posner, 2012; Posner, 1980).

Initial findings from studies using the CAT have provided promising construct-relevant evidence. For instance, Hoyer et al. (2021) demonstrated significant developmental changes in distractibility throughout childhood and adolescence, consistent with the maturation of neural networks involved in attention and motor control. Extending this lifespan perspective, Hoyer et al. (2023a) reported that specific distractibility components, such as arousal-driven facilitation, differ in older adults, paralleling known age-related declines in cortical network integrity and inhibitory control (ElShafei et al., 2020a, 2022). Together, these findings provide construct-relevant validity evidence for the CAT, suggesting that it can dissociate meaningful attentional processes (e.g., voluntary orienting, distractor-driven capture, and motor impulsivity) and track their evolution across the lifespan. This construct-relevant validity suggests potential relevance for clinical profiling and longitudinal monitoring.

Other psychometric properties are crucial to assess a test validity. Indeed, internal consistency provide evidence for convergence among closely related indices (e.g., distinct measures of impulsivity) and differentiation between separate constructs (e.g., attentional capture versus voluntary orienting; Streiner et al., 2015). Test–retest reliability is also critical, particularly in developmental and clinical contexts involving repeated assessments to monitor disease progression, maturational trajectories, or treatment efficacy (Weir, 2005). For instance, test–retest reliably distinguishing genuine therapeutic effects, such as reduced impulsivity or improved resistance to distraction in ADHD, from random measurement fluctuations requires stable pre- and post-intervention measures (Conners et al., 2018). Similarly, inter-rater (i.e., between-examiner) reliability is essential when multiple examiners (clinicians, graduate students, or research assistants) administer the same test, ensuring consistent scoring and interpretation, thereby minimizing examiner variability that could mask genuine individual differences (McHugh, 2012; Streiner et al., 2015).

Validating the CAT would advance research by providing a precise tool to measure how unexpected stimuli influence performance in realistic contexts. Methodologically, the CAT could clarify the relationship between attentional behavior and neural dynamics, particularly activity within the fronto-parietal networks known to underpin attentional control (Corbetta and Shulman, 2002; Petersen and Posner, 2012; Wetzel and Schröger, 2014). Furthermore, previous electroencephalography (EEG) and magnetoencephalography (MEG) research using the CAT has highlighted how evoked responses and alpha- and gamma-band activities within these networks change during voluntary attention orienting and distraction by irrelevant sounds (ElShafei et al., 2018, 2020b, 2022, Bidet-Caulet et al., 2015).

This paper reports two experiments designed to substantiate the CAT’s psychometric properties. Experiment 1 assesses internal consistency and inter-rater reliability. Experiment 2 evaluates short-term test–retest stability, as well as convergent and divergent validity, through comparison with established attention tasks (TAP-M, KiTAP) and general cognitive measures (Wechsler intelligence scales), respectively. Rather than structural modeling, we adopt a construct-relevant validation approach based on observed-score coherence and theoretically guided associations.

In line with psychometric validation standards, we formulated specific hypotheses for each aspect of the CAT’s construct-relevant validity. (1) We hypothesized adequate *internal consistency*, expecting positive correlations between indices measuring similar attentional constructs or measures (e.g., arousal, sustained attention) and minimal correlations between indices assessing distinct constructs (e.g., attentional capture versus voluntary orienting). (2) Minimal *inter-rater effect*, consistent with procedural robustness, was also predicted. (3) We anticipated strong *test–retest reliability*, with no evidence for session-to-session differences in core behavioral indices, thus reflecting temporal stability. Finally, (4) meaningful *concurrent validity* was hypothesized, including both convergent and divergent components. Specifically, we expected evidence for positive correlations between CAT indices and corresponding measures from established attention batteries (KiTAP, TAP-M), demonstrating convergent validity. Divergent validity, on the other hand, would show no evidence for correlations between CAT scores and unrelated cognitive abilities assessed by verbal reasoning and abstract problem-solving subtests from the Wechsler intelligence scales (e.g., Similarities, Matrix Reasoning). By confirming that the CAT yields consistent and interpretable indices of attentional control, this work aims to support its integration into research and potential clinical assessment.

## 2 Methods

### 2.1 Stimuli and task

**Figure 1:**
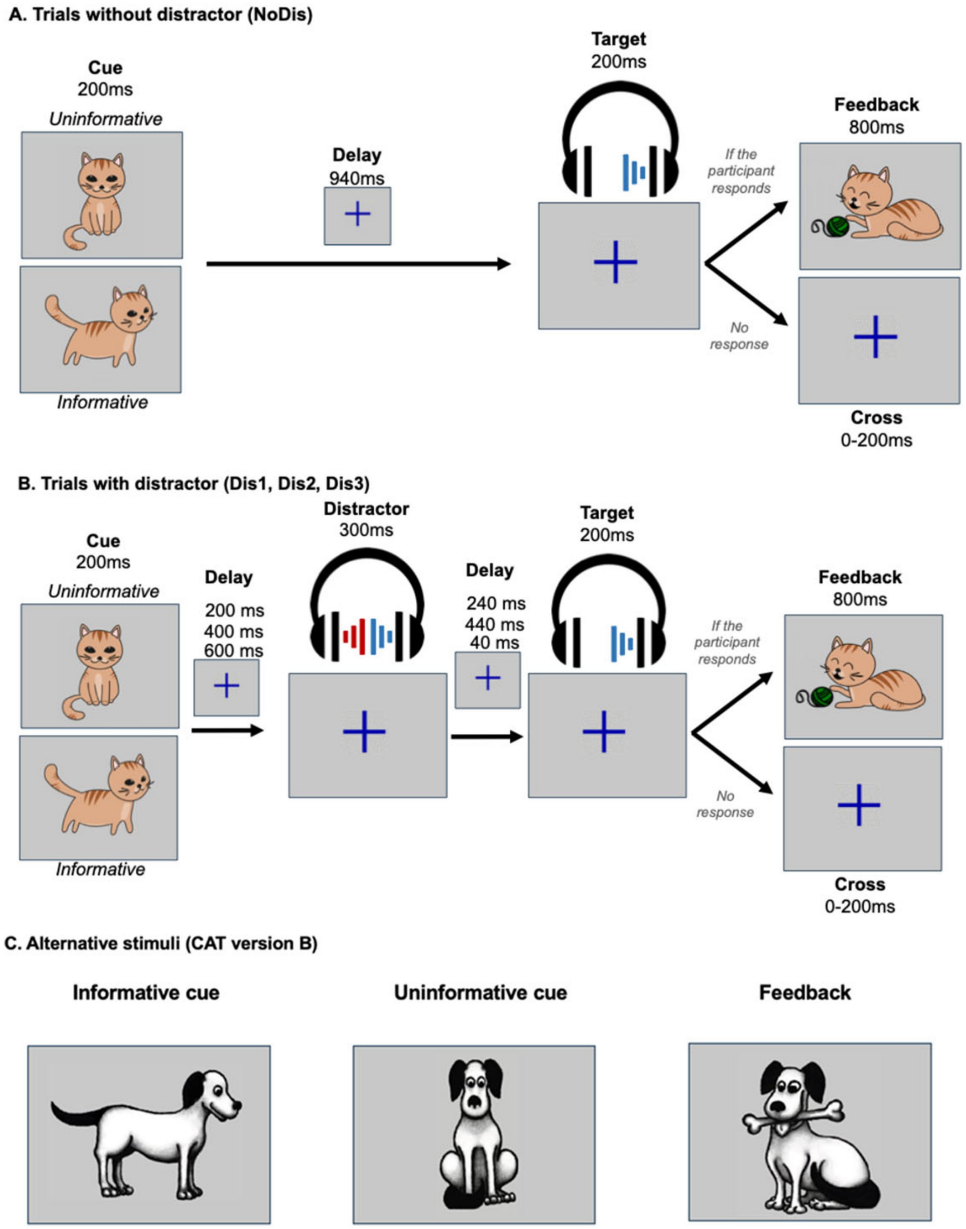
Task protocol and trial visualization for the CAT. **(A)** In uninformative trials, a facing-front cat was used as a visual cue (200 ms duration), indicating that the target sound would be played in either the left or right ear. In informative trials, a facing left or right cat visual cue (200 ms duration) indicated in which ear (left or right, respectively) the target sound would be played (200 ms duration) after a delay (940 ms). If the participant gave a correct answer, feedback (800 ms duration) was displayed. **(B)** In trials with a distractor, the task was similar, but a binaural distracting sound (300 ms duration), such as a phone ring, was played during the delay between cue and target. The distracting sound could equiprobably onset at three different times: 200 ms, 400 ms, or 600 ms after the cue offset. **(C)** Shows alternate stimuli images for the CAT version B, using a dog instead of a cat.

The CAT stimuli and task structure are described in detail in Hoyer et al. (2021, 2023a). Briefly, half of the trials (50%) began with a 200-ms visual cue depicting a dog facing left, right, or forward on a gray background at screen center. An alternative version used a cat instead of a dog. After a 940-ms delay, participants heard a 200-ms monaural target sound (dog barking) presented at 15 dB SL (~43 dBA) via headphones. The other half followed the same structure but included a binaural distractor sound (35 dB SL, 300 ms) during the delay. Eighteen distractor sounds (e.g., ringtones, alarms) occurred equally often at three possible timings after cue offset (200 ms: Dis1, 400 ms: Dis2, or 600 ms: Dis3), as illustrated in Figure 1. Each experimental block included 24 no-distractor trials (NoDis) and 24 distractor trials (8 per distractor timing). Over three blocks, participants thus completed 72 NoDis trials and 24 trials per distractor condition. Cue direction and target side (left/right) were balanced across all trial types. In 75% of trials (informative condition), the dog faced left or right, reliably indicating the ear in which the target bark would be presented (37.5% left, 37.5% right). In the remaining 25% of trials (uninformative condition), the dog faced forward, providing no indication of target side (12.5% left, 12.5% right). Each distractor sound was presented four times across the entire experiment, never exceeding two repetitions per block, to prevent habituation. Participants were instructed to press a key as quickly as possible upon detecting the target sound. On informative trials, they were explicitly told to focus on the ear indicated by the dog’s orientation, which was always accurate. They were informed that distractors might occasionally occur but instructed to respond promptly regardless. When no visual cue appeared, a blue fixation cross was displayed at screen center, and participants were asked to maintain gaze on it. During each trial, if participants responded within 3,300 ms after target onset, positive visual feedback (example: a cat playing with a ball of yarn; see Figure 1) appeared for 800 ms. Following a 500-ms delay, a fixation cross was displayed for a randomized interval (1,700–1,900 ms). If participants failed to respond within 3,300 ms, the fixation cross remained on-screen an additional 100–300 ms. After each block, participants received feedback about their average reaction time to encourage rapid responses.

Before the main task, participants completed a brief training block of 10 trials following the timing and structure of the experimental trials. Two of these training trials included distractors randomly selected from the same pool of 18 sounds used later. To ensure participants understand the instructions, the training block was repeated until 7 hits out of the 10 trials was reached.

### 2.2 Common CAT administration procedure

**Figure 2:**
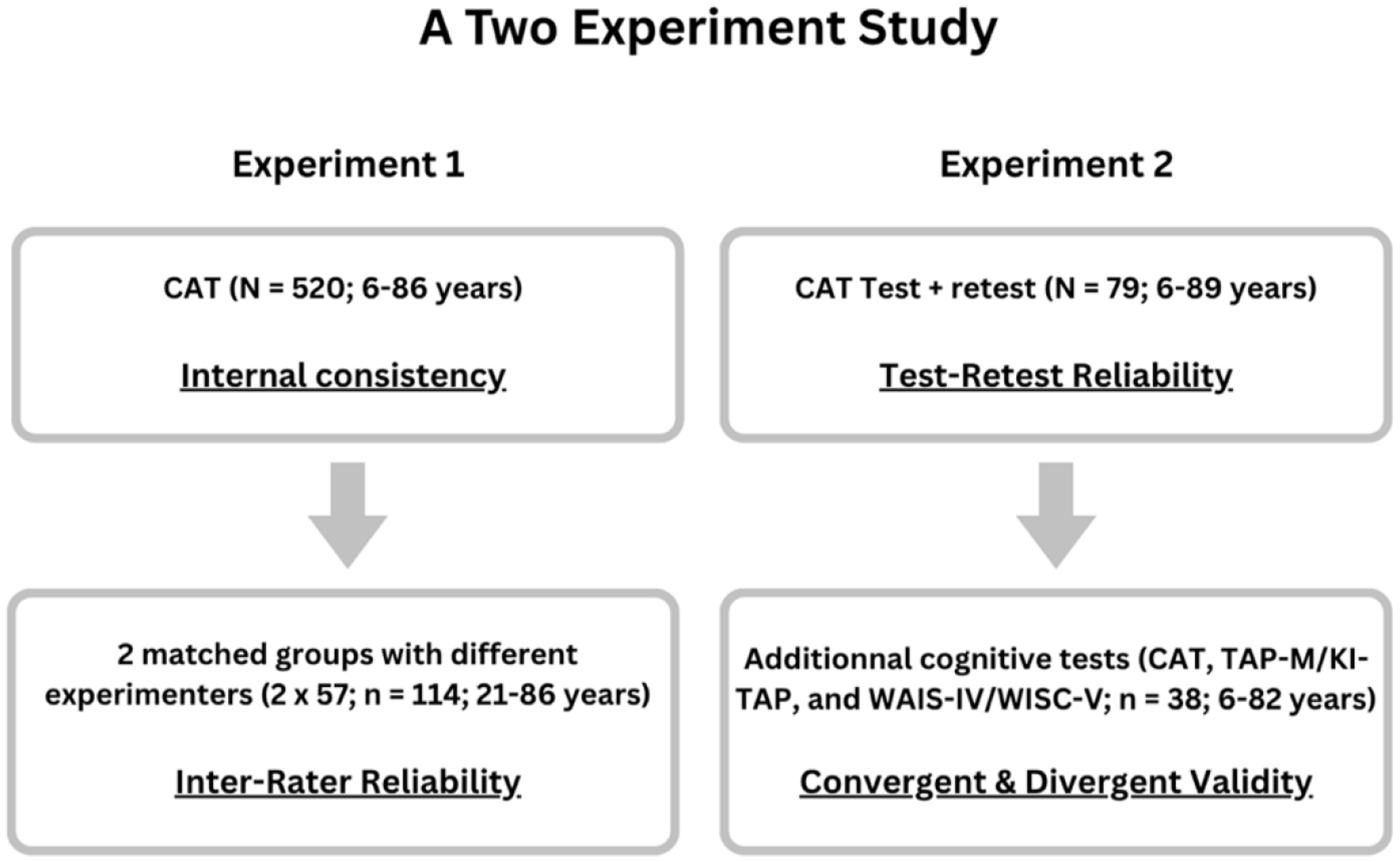
Schematic overview of the two-experiment design, sample sizes, and psychometric objectives. Experiment 1 examined internal consistency (*N =* 520; ages 6–86 years) and inter-rater reliability (*N =* 114; ages 21–86 years), the latter based on two matched groups of 57 participants assessed by two different experimenters. Experiment 2 examined test–retest reliability (*N =* 79; ages 6–89 years) and convergent and divergent validity in a subsample of participants who completed a longer assessment protocol (*N =* 38; ages 6–82 years), including external attention and cognitive measures (TAP-M or KiTAP, and Wechsler intelligence scales). CAT, Competitive Attention Test; TAP-M, Test of Attentional Performance for adults; KiTAP, Test of Attentional Performance for children; WAIS-IV, Wechsler Adult Intelligence Scale-Fourth Edition; WISC-V, Wechsler Intelligence Scale for Children-Fifth Edition.

The CAT was administered using the same standardized procedure in both experiments, following the protocol previously described in Hoyer et al. (2021, 2023a). Figure 2 illustrates the overall study design, sample sizes, and allocation of participants across the two experiments, as well as psychometric analyses. Participants completed the task in small groups of two to four individuals. Adults were assessed either in the laboratory or at the university, while children were tested in a quiet room at their school. Participants sat approximately 50 cm from a laptop presenting stimuli and recording behavioral responses via Presentation software (Neurobehavioral Systems, Albany, CA). All auditory stimuli were delivered through headphones at individually adjusted volumes, established beforehand by measuring auditory thresholds using the Bekesy tracking method.

After verbal instructions, participants first completed a brief training phase, followed by three experimental blocks of 48 pseudorandomized trials, each lasting about 4 min. The block order was randomized across participants. Sessions typically lasted around 45 min for children and 30 min for adults. The CAT was administered by different experimenters, all trained neuropsychology students.

### 2.3 Measurements and parameters

The fourteen CAT indices are labelled descriptively (e.g., “Median RT,” “Cue Responses,” “Arousal”), each reflecting a specific cognitive component such as processing speed, impulsivity, attentional capture, or sustained attention. Abbreviations and definitions for all indices are summarized in Table 1.

**Table 1:** CAT indices, definitions, and neuropsychological interpretations used across experiments 1 and 2.

Measure name	Description	Neuropsychological index
Median RT	Median Positive reaction times (ms).	Index of long-term sustained attention
Mean SDRT	RT standard deviation (ms): mean standard deviation of RT + in the NoDis condition.	Variability in processing speed: Long-term sustained attention.
Orienting	Defined by (medianRTNoDisUninf – medianRTNoDisInf) / medianRTAll	Voluntary attention orienting
Arousal	Phasic arousal effect defined by (medianRTNoDis – medianRTDis1) / medianRTAll	Phasic arousal due to a distractor
Capture	Distraction effect defined by (medianRTDis3 – medianRTDis1) / medianRTAll.	Attention capture
Correct Responses	Correct Response (CorRep, %): the percentage of correct responses.	Index of general performance.
Cue Responses	Cue response (CueRep, %): the percentage of responses performed during the 150–450 ms period post-cue onset.	Index of reactive impulsivity to relevant stimuli.
Distractor Responses	Distractor response (DisRep, %): the percentage of responses performed during the 150–450 ms period post-distractor onset.	Index of reactive impulsivity to irrelevant stimuli.
Random Responses	Random response (RandRep, %): the percentage of responses performed during the remaining periods of the trials.	Index of motor control in absence of stimuli.
Anticipated Responses	Anticipated response (AntRep, %): the percentage of responses anticipated in NoDis conditions before the target stimulus.	Index of proactive impulsivity to relevant stimuli.
Late Responses (no-distractor)	Late response (LateRep, %): the percentage of late responses performed in the NoDis condition.	Index of attentionnal lapses.
Late Responses (contrast)	Late response (LateRep, %): the contrast of the percentage of late responses performed in the Dis condition against the NoDis condition.	Index of attentionnal lapses.
Missed Responses (no-distractor)	Missed response (MissRep, %): the percentage of trials without any response made during the entire trial duration up to 3,300 ms posttarget in the NoDis condition.	Index of attentionnal lapses.
Missed Responses (contrast)	Missed response (MissRep, %): the contrast of the percentage of missed responses performed in the Dis condition against the NoDis condition.	Index of attentionnal lapses.

NoDis: Trials without a distractor. Dis: Trials with a distractor, categorized by onset latency relative to the cue (Dis1: short delay, Dis2: medium delay, Dis3: long delay). NoDisUninf: No-distractor trials with an uninformative cue. NoDisInf: No-distractor trials with an informative cue.

### 2.4 General statistical approach

We adopted a Bayesian statistical framework because validation studies often require quantifying evidence in favor of stability or absence of effects, rather than merely testing for differences (Jeffreys, 1998; Rouder et al., 2017). Bayesian analyses allow direct comparison of alternative and null hypotheses through Bayes Factors (BF₁₀), which is particularly informative when evaluating reliability and construct coherence, where evidence supporting the null hypothesis is theoretically meaningful (Cohen, 1994; Masson, 2011; Wagenmakers, 2007; Wagenmakers et al., 2011). All analyses employed the standard two-tailed Jeffreys-Zellner-Siow Cauchy prior (scale r = 0.707).

We report *BF₁₀* values, interpreted according to Wagenmakers (2007): *BF₁₀* above 3 indicates moderate evidence, above 10 indicates strong evidence, above 30 very strong evidence, and BF_10_ above 100 indicates extreme evidence supporting the alternative hypothesis. Conversely, *BF₁₀* values below 1 indicate increasing support for the null hypothesis: between 1 and 1/3 anecdotal, below 1/3 moderate, and below 1/10 strong evidence against the alternative hypothesis. In correlational analyses, the *BF₁₀* indicates how likely it is that this observed relationship is genuine while the Kendall’s *τ* is interpreted as an ordinal effect size reflecting the strength of monotonic associations.

Given that age-related behavioral effects on CAT performance have been characterized in previous studies, age was included as a covariate solely to partial out global age-related variance in attentional performance and was treated as a nuisance variable rather than as a factor of theoretical interest. Accordingly, the present analyses do not aim to re-examine developmental trajectories or to formally test age-related measurement invariance.

### 2.5 Experiment 1—internal consistency and inter-rater reliability

#### 2.5.1 Participants

For internal consistency, we used data from a combined sample of two previous studies employing the CAT (Hoyer et al., 2021, 2023a). All participants (*N =* 520) were French-speaking, aged 6–86 years, with normal or corrected-to-normal hearing and vision, no known neurological or psychiatric disorders, and had not taken medication affecting the central nervous system within the 24 h prior to testing (Tables 2A,B). Adults provided written informed consent, while minors gave verbal assent along with their parents or guardians written consent. This study was conducted according to the Helsinki Declaration, Convention of the Council of Europe on Human Rights and Biomedicine, and the experimental paradigm was approved by the French ethics committee Comité de Protection des Personnes (approval number 2014-A01289-38). To assess inter-rater effects, we restricted the analysis to a subset of 114 participants, as reliable matching between participants and experimenters was only possible for 57 pairs. This resulted in two matched adult groups (*n =* 57 each), equated on age, gender, and laterality, and assessed by two different experimenters (see Table 2B). Other experimenters had tested fewer than 15 participants (insufficient statistical power). Matching was performed using the *MatchIt* package in R (*matchit* function, nearest neighbour algorithm, caliper = 0.20), resulting in two subsamples with comparable age distributions.

**Table 2:** Sample characteristics for internal consistency, inter-rater reliability, test–retest, and convergent and divergent validity analyses.

(A)			
Internal consistency – total sample (*N =* 520)			
Age	M	24.59	
	SD	20.67	
Age range (*N*)	6–7	45	
	8–9	52	
	10–11	61	
	12–13	53	
	14–15	49	
	16–17	46	
	18–20	28	
	21–25	32	
	26–30	24	
	31–40	25	
	41–50	27	
	51–60	25	
	61–70	25	
	71–89	28	
Laterality (*N*)	Ambidextrous	10	
	Right	457	
	Left	53	
Biological sex (%)	Male	232	
	Female	288	

(B)				
Inter-rater reliability—matched subsamples by experimenters (*n =* 114)				
	Experimenter 1		Experimenter 2	
Age	M	59.21	M	49.91
	SD	19.64	SD	19.68
	21–25	8	21–25	8
	26–30	4	26–30	4
	31–40	9	31–40	9
	41–50	9	41–50	9
	51–60	8	51–60	8
	61–70	9	61–70	9
	71–89	10	71–90	10
Laterality (*N*)	Ambidextrous	1	Ambidextrous	1
	Right	54	Right	54
	Left	2	Left	2
Biological sex (%)	Male	17	Male	17
	Female	40	Female	40

(C)		
Test–retest reliability—total sample (*N =* 79)		
Age	M	27.71
	SD	20.85
Age range (N)	6–7	6 (7.6%)
	8–9	6 (7.6%)
	10–11	8 (10.1%)
	12–13	4 (5.1%)
	14–15	6 (7.6%)
	16–17	6 (7.6%)
	18–20	7 (8.9%)
	21–25	6 (7.6%)
	26–30	3 (3.8%)
	31–40	7 (8.9%)
	41–50	5 (6.3%)
	51–60	7 (8.9%)
	61–70	4 (5.1%)
	71–89	4 (5.1%)
Laterality (N)	Right	66 (83.5%)
	Left	13 (16.5%)
Biological sex (%)	Male	32 (50.5%)
	Female	47 (59.5%)

(D)		
Convergent and divergent validity—total sample (*n =* 38)		
Age	M	26.74
	SD	20.86
Age range (*N*)	6–7	3 (7.9%)
	8–9	3 (7.9%)
	10–11	4 (10.5%)
	12–13	3 (7.9%)
	14–15	2 (5.3%)
	16–17	3 (7.9%)
	18–20	3 (7.9%)
	21–25	4 (10.5%)
	26–30	1 (2.6%)
	31–40	3 (7.9%)
	41–50	2 (5.3%)
	51–60	4 (10.5%)
	61–70	1 (2.6%)
	71–89	2 (5.3%)
Laterality (N)	Right	33 (86.8%)
	Left	5 (13.2%)
Biological sex (%)	Male	13 (34.2%)
	Female	25 (65.8%)

Panels A–D present sample characteristics for the internal consistency, inter-rater reliability, test–retest reliability, and convergent/divergent validity analyses, respectively. For this table, data have been pooled across age ranges to provide an overall view of participant distribution.

#### 2.5.2 Statistical analyses

*Internal consistency* (*N =* 520, 6–86 years). Internal consistency was assessed with partial Bayesian Kendall correlations controlled for age using the *correlation*() function in R (v 4.3.2; *BayesFactor* package). The resulting statistics are reported as Kendall’s τ and the corresponding BF₁₀. This analytic strategy verifies that indices intended to capture the same attentional facet cluster together and that those indexing distinct facets remain orthogonal. Random response rates were excluded from these analyses because the distribution was highly zero-inflated, resulting in insufficient variability to support stable correlation estimates.

#### 2.5.3 Inter-rater reliability (*N =* 114, 21–86 years)

Because each participant was assessed by a single experimenter, experimenter effects could not be evaluated using within-subject agreement metrics. Bayesian between-group linear models were used to test for experimenter effects. Intraclass correlation coefficients were not applicable, as they require the same participants to be evaluated by multiple experimenters. In this context, evidence for the absence of experimenter effects provides support for the reproducibility of CAT scores across experimenter. Accordingly, between-group comparisons were conducted using lmBF(), with Experimenter entered as a fixed factor and no age covariate included. Under consistent administration, no systematic differences between experimenter groups are expected.

### 2.6 Experiment 2—reliability and concurrent validity

#### 2.6.1 Participants

Seventy-nine French-speaking volunteers aged 7–89 years took part in experiment 2. The study followed a test–retest design with an interval of approximately 3 weeks (*M* = 26.16 days, *SD* = 11.39). All participants (*N =* 79, see Tables 2C,D for sociodemographics) completed the CAT at baseline (Session 1) and again after 3 weeks (Session 2). Inclusion criteria and consent collection method were identical to Experiment 1. Adults were compensated 10 euros per hour – children did not receive any compensation. This study was approved by the French ethics committee Comité de Protection des Personnes (approval number 2018-A02597-48).

#### 2.6.2 Procedure specificities

Participants completed two experimental sessions. In one session, they performed the CAT-A version, which used dogs as visual cues and feedback and a dog bark as the target sound (Hoyer et al., 2021, 2023a). In the other session, they performed the CAT-B version, in which cats replaced dogs as visual stimuli, feedback, and target sounds. Apart from these stimulus substitutions, all task parameters, including stimulus duration and intertrial intervals, were identical across versions. Session order was counterbalanced across participants. Each session began with brief instructions and a short training phase, followed by three blocks of 48 pseudorandomized CAT trials.

A subgroup (*N =* 38) performed additional tests during the first session. After completing the CAT, adults and children performed, the TAP-M or KiTAP subtests (Distractibility, Go/NoGo, Sustained Attention). Since the KiTAP and TAP-M are only normed for ages 6–9 and 18–89, we only analyzed raw scores from those tests. Participants aged 10–14 completed the KiTAP, 15 years and older the TAP-M. Fifteen years was chosen as the cutoff based on previous literature suggesting that sustained attention stabilizes around 15 years of age (e.g., Hoyer et al., 2021; Lin et al., 1999; Thillay et al., 2015).

Finally, children aged 6–16 completed subtests from the Wechsler Intelligence Scale for Children (WISC-V); while participants aged 17 and older completed subtests from the Wechsler Adult Intelligence Scale (WAIS-IV). The subtests administered were Similarities, Matrix Reasoning, Coding, and Symbol Search, which, respectively, measure verbal reasoning, fluid intelligence, and processing speed. Standardized scores from these tests were extracted and analyzed.

#### 2.6.3 Statistical analysis

##### 2.6.3.1 Test–retest reliability (*N =* 79, 6–89 years)

We assessed test–retest reliability using Bayesian ANCOVAs with Session (1 vs. 2) as a within-subject factor and Age as a covariate (lmBF() R function from the BayesFactor package). The aim was to assess the stability of CAT scores across the three-week interval by testing for equivalence of score distributions across sessions. For each CAT index, BF₁₀ quantified evidence for the main effects of Session, Age, and the Session × Age interaction. Strong test–retest reliability was indicated by the absence of a session effect (*BF₁₀* < 1 for the Session term).

##### 2.6.3.2 Concurrent validity (*N =* 38, 6–82 years)

We again used partial Bayesian Kendall correlations (controlling for age). Convergent validity was assessed by correlating relevant CAT indices with attentional measures from the TAP-M or KiTAP battery. Divergent validity was evaluated by correlating CAT indices with broader cognitive measures (Similarities and Matrix Reasoning subtests). Although attentional resources contribute partially to all Wechsler subtests, we predicted weaker associations with subtests focusing on reasoning and verbal abstraction compared to those specifically targeting attentional control (Mayes and Calhoun, 2006). Note that two subjects were lost from the initial 40 participants because of recording errors: one for the KiTAP Distractibility task and one for the TAP-M Go/NoGo task.

## 3 Results

We expected the CAT to demonstrate good internal consistency, high inter-rater and test–retest reliability, as well as convergent validity with attention-specific measures (TAP-M, KiTAP) and divergent validity with domain-general cognitive subtests (WISC/WAIS). Given the large number of variables tested, this section only addresses key patterns and effects; all correlation coefficients, Bayes Factors, and detailed statistics are presented in the accompanying tables.

### 3.1 Experiment 1

#### 3.1.1 Internal consistency (*N =* 520; 6–86 years)

**Figure 3:**
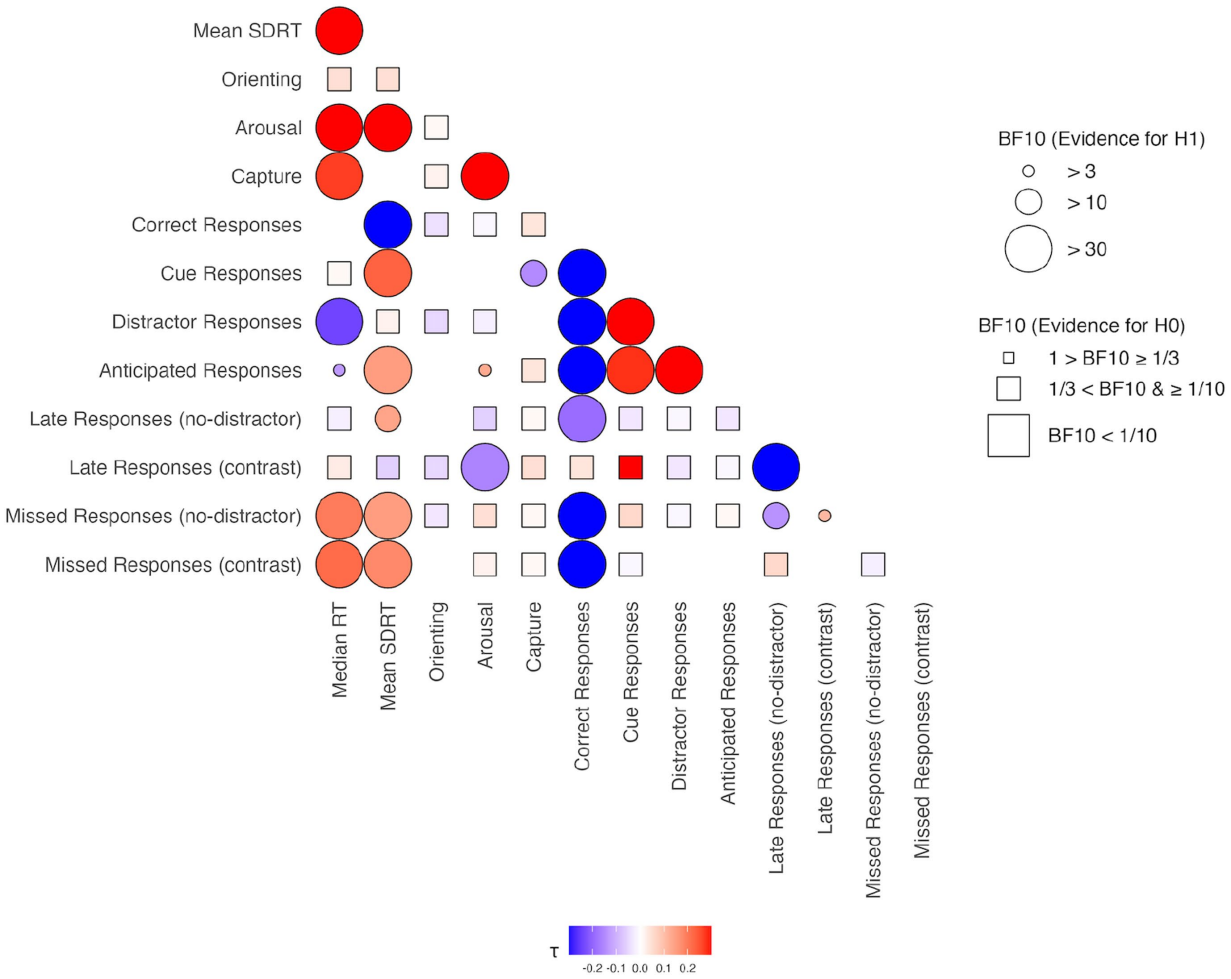
Internal consistency: partial Bayesian correlation matrix for CAT measures. All correlations are Kendall’s *τ*, residualized for age and shown with color (blue for negative values and red for positive values). BF₁₀ values, shown with shape sizes, indicate the strength of evidence for an association. The squares indicate support for the H1 hypothesis (correlation between the measures), while the circles symbolize support for the H0 hypothesis (no correlation). Absence of shapes indicates no conclusive result.

To evaluate the internal consistency of the CAT, partial Bayesian correlations were computed among its behavioral indices. Measures targeting the same construct were expected to correlate positively (e.g., Anticipated and Distractor Responses for impulsivity), while opposing or unrelated constructs were expected to show negative or no association. See Table 3 and Figure 3 for results and reported values of *BF₁₀* values and Kendall’s *τ*.

**Table 3:** Internal consistency: partial Bayesian correlation matrix for the CAT measures in children and adults.

Measures	Median RT	Mean SDRT	Orienting	Arousal	Capture	Correct responses	Cue responses	Distractor responses	Anticipated responses	Late responses (no-distractor)	Late responses (contrast)	Missed responses (no-distractor)
Mean SDRT	5.87e+79 ≠											
Orienting	0.19 =	0.19 =										
Arousal	2.27e+13 ≠	6.00e+10 ≠	0.11 =									
Capture	2.22e+07 ≠	0.57	0.12 =	8.713 + 45 ≠								
Correct Responses	0.61	4.46e+12 ≠	0.16 =	0.10 =	0.15 =							
Cue Responses	0.10 =	1.64e+05 ≠	1.30	1.17	28.44 ≠	1.35e+25 ≠						
Distractor Responses	7.09e+05 ≠	0.11 =	0.18 =	0.12 =	0.69	1.93e+44 ≠	2.61e+28 ≠					
Anticipated Responses	6.73 ≠	36.53 ≠	0.39	7.11 ≠	0.14 =	4.37e+51 ≠	2.58e+08 ≠	2.42e+34 ≠				
Late Responses (no-distractor)	0.11 =	27.70 ≠	0.61	0.23 =	0.11 =	1301.39 ≠	0.14 =	0.10 =	0.12 =			
Late Responses (contrast)	0.12 =	0.31 =	0.18 =	70.08 ≠	0.19 =	0.14 =	0.24 =	0.12 =	0.11 =	4.22e+67 ≠		
Missed Responses (no-distractor)	6.38e+03 ≠	37.94 ≠	0.14 =	0.20 =	0.10 =	3.62e+25 ≠	0.26 =	0.11 =	0.10 =	15.40 ≠	3.56 ≠	
Missed Responses (contrast)	4.73e+04 ≠	1032.50 ≠	0.62	0.11 =	0.10 =	1.48e+14 ≠	0.10 =	0.55	1.85	0.28 =	2.11	0.11 =

BF₁₀ for correlations, residualized for age and shown in Figure 3. BF₁₀ values indicate the strength of evidence for an association. The ≠ symbol indicates support for the H_1_ hypothesis (correlation between the measures), while the = symbolizes support for the H_0_ hypothesis (no correlation).

Mean SDRT showed a strong positive correlation with Median RT and smaller, though still well-supported, positive correlations with Anticipated Responses, Cue Responses, Missed Responses in both no-distractor and contrast conditions, as well as Late Responses when no distractor was present. Arousal correlated positively with Median RT, Mean SDRT, and very strongly with Capture. In contrast, higher arousal predicted fewer Late Responses when distractors were contrasted with no-distractor trials. Capture itself correlated positively with Median RT and negatively with cue-driven responding. Orienting, by comparison, exhibited no correlations with other indices (most *BF₁₀* < 0.33, all *BF₁₀* < 1.30), indicating little overlap with the other CAT constructs.

Response accuracy patterns were also highly coherent: Correct Responses were inversely correlated with multiple error types, namely Distractor Responses, Anticipated Responses, Late Responses without distractors, and Missed-Response metrics. Error measures were inter-related: Anticipated and Distractor Responses covaried positively and each aligned with Cue Responses, while not covarying with Miss and Late Response metrics, illustrating a coherent specific impulsivity-interference cluster.

Taken together, these correlations substantiate the internal structure of the CAT: variability and speed indices converge, arousal and capture track shared attentional engagement, and accuracy measures oppose diverse error profiles. Associations with *BF₁₀* between 3 and 30 warrant cautious interpretation, yet the overall pattern is theoretically consistent.

##### 3.1.1.1 Inter-rater reliability (*n =* 114, 21–86 years)

We next assessed inter-rater reliability. Bayesian between-group linear models were used to test for inter-rater (see Table 4 for reported values of tests results, mean, and *SD*) provided moderate evidence for an experimenter effect on Mean SDRT and anecdotal evidence on SDRT (no-distractor Block 1), Late Responses (no-distractor), and Late Responses (no-distractor Block 1). In every case, values were higher for Experimenter 2, indicating slightly greater variability and delayed responding in that subsample. All remaining CAT indices yielded *BF₁₀* < 1, indicating no meaningful experimenter differences and overall good inter-rater reliability.

**Table 4:** Inter-rater reliability: Bayesian between-group linear models results for the CAT measures.

Measures	Experimenter 1		Experimenter 2		BF10	error %
	M	SD	M	SD		
Median RT	386.2	104.2	399.7	114.7	0.242 =	0.03
Mean SDRT	103.4	38.2	124.2	50.6	3.007 ≠	0.01
SDRT (no-distractor block 1)	106.9	47.0	137.9	83.7	2.765	0.01
SDRT (no-distractor block 2)	103.6	49.0	125.9	75.7	0.936	0.01
SDRT (no-distractor block 3)	99.6	44.1	108.7	40.7	0.361	0.02
Orienting	4.1	35.2	6.8	39.7	0.213 =	0.03
Arousal	83.4	72.9	84.3	54.6	0.199 =	0.03
Capture	86.7	47.2	76.0	46.0	0.389	0.02
Correct responses	88.8	9.1	86.0	9.4	0.620	0.02
Cue responses	0.1	0.3	0.3	0.8	0.676	0.02
Random responses	0.0	0.2	0.2	0.7	0.601	0.02
Distractor responses	2.2	2.6	2.9	3.6	0.406	0.02
Anticipated responses	1.4	2.2	1.6	2.2	0.215 =	0.03
Late response (no-distractor)	8.8	5.2	10.7	5.2	1.166	0.01
Late response (no-distractor block 1)	11.1	8.1	15.0	12.3	1.147	0.01
Late response (no-distractor block 2)	7.9	6.9	9.9	6.4	0.659	0.02
Late response (no-distractor block 3)	7.4	6.9	7.3	5.7	0.200 =	0.03
Missed response (no-distractor)	2.7	9.0	3.4	7.8	0.213 =	0.03
Missed responses (contrast)	−0.2	2.5	−0.7	2.1	0.322 =	0.03

Means (M) and SDs are shown for each experimenter; BF₁₀ values indicate the strength of evidence for an association. The ≠ symbol indicates support for the H_1_ hypothesis (effect of experimenters), while the = symbolizes support for the H_0_ hypothesis (no effect of experimenters).

### 3.2 Experiment 2

#### 3.2.1 Test–retest reliability (*N =* 79, 6–89 years)

We assessed the test–retest reliability of CAT indices using Bayesian ANCOVAs with Session (1 vs. 2) as a within-subject factor and Age as a covariate.

**Figure 4:**
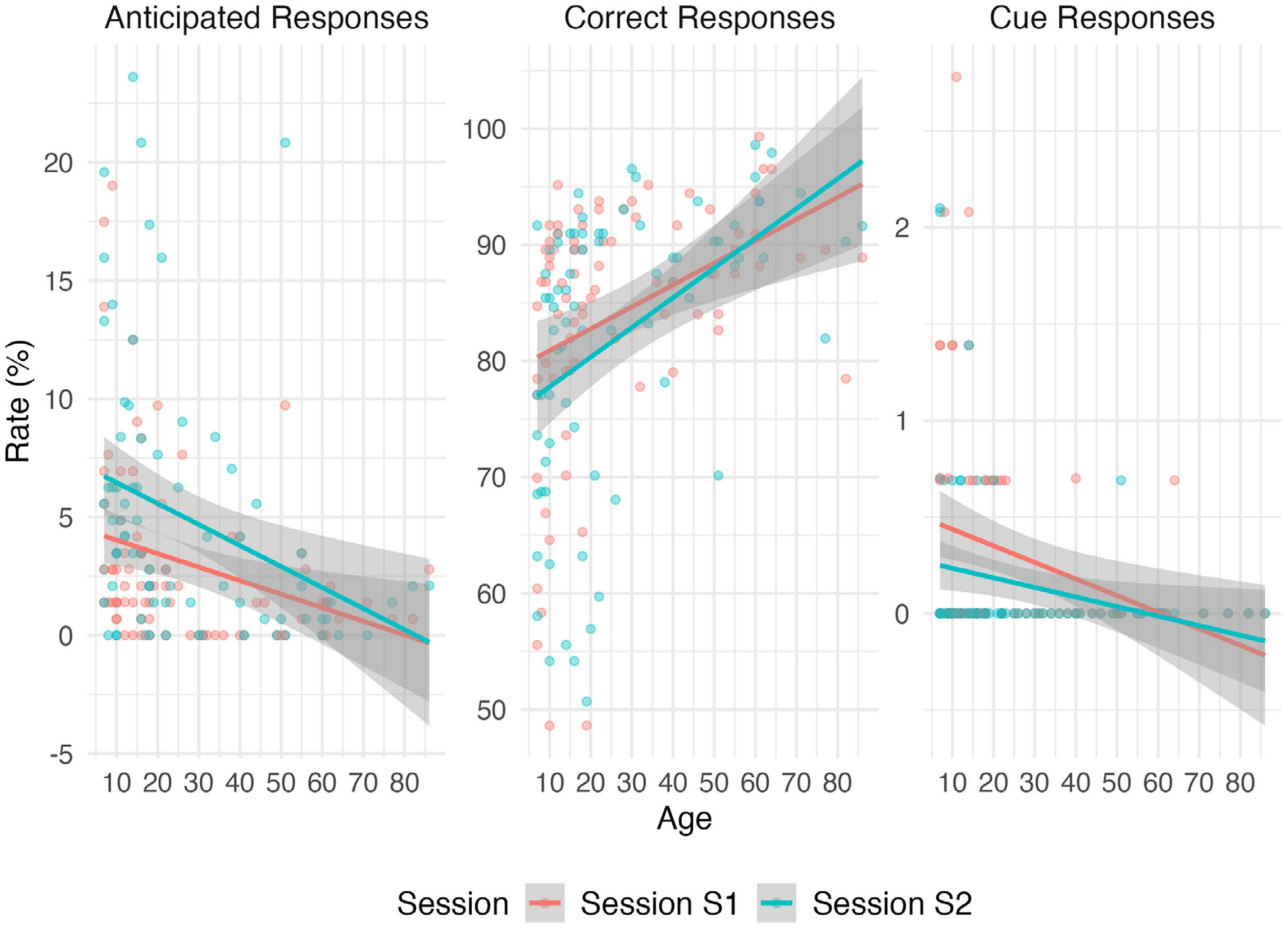
Age-dependent session effects on correct, cue, and anticipated responses. Regression lines (±95% CI) show model fits for each session. Consistent with the Bayesian ANCOVA results, evidence for session × age interactions was observed for correct, cue, and anticipated response rates.

As shown in Table 5, evidence for no main effect of Session was found for eight indices (*BF₁₀* < 0.33: Orienting, Capture, Correct, Random, Distractor, Late in no-distractor, Missed in no-distractor, and contrast Responses). No conclusive evidence was found for the other six indices (0.33 < *BF₁₀* < 3: Median RT and Mean SDRT, Arousal, Cue, Anticipated and Late in contrast Responses). Standardized effect sizes quantifying the age-adjusted Session-related mean shift (Cohen’s d) are reported in Supplementary Table 1. As expected, Age had a substantial effect on several measures (e.g., Correct Responses; Anticipated Responses), consistent with known developmental trajectories of attention (Hoyer et al., 2021, 2023a). Notably, evidence for Session by Age interaction effects was observed for Correct Responses, Cue Responses, and Anticipated Responses, indicating that short-term changes in performance varied by age, with younger participants tending to show larger increases in impulsive responses on retest (see Figure 4).

**Table 5:** Test–retest reliability: Bayesian ANCOVA results for the CAT measures.

Measures	Session		Age		Session × age	
	BF10	Error %	BF10	Error %	BF10	Error %
Median RT	0.702	0.02	0.191 =	< 0.01	0.130 =	1.40
Mean SDRT	1.157	0.01	1.156	< 0.01	1.423	0.91
Orienting	0.227 =	0.04	0.171 =	< 0.01	0.039 =	1.11
Arousal	1.466	0.01	0.194 =	< 0.01	0.283	1.83
Capture	0.171 =	0.05	1.309	< 0.01	0.220 =	1.48
Correct Responses	0.292 =	0.04	144187.300 ≠	0.01	45347.090 ≠	0.86
Cue Responses	0.716	0.02	82.025 ≠	0.01	63.982 ≠	1.02
Random Responses	0.195 =	0.05	14.540 ≠	0.01	2.870	2.15
Distractor Responses	0.171 =	0.05	1.248	< 0.01	0.210 =	1.50
Anticipated Responses	2.800	0.01	329.749 ≠	0.01	1214.693 ≠	1.36
Late Responses (no-distractor)	0.189 =	0.05	0.309 =	< 0.01	0.067 =	12.37
Late Responses (contrast)	0.346	0.03	0.227 =	< 0.01	0.793	1.07
Missed Responses (no-distractor)	0.203 =	0.05	12.298 ≠	< 0.01	2.872	10.32
Missed Responses (contrast)	0.230 =	0.04	0.221 =	< 0.01	0.049 =	2.37

BF₁₀ values quantify evidence for Session, Age, and Session × Age effects. The ≠ symbol indicates support for the H_1_ hypothesis (factor effect), while the = symbolizes support for the H_0_ hypothesis (no factor effect).

Together, these results support acceptable short-term test–retest reliability for most CAT measures, while highlighting specific indices (particularly error-type responses) that are more sensitive to repetition effects in younger individuals.

#### 3.2.2 Concurrent validity (*n =* 38)

##### 3.2.2.1 Convergent validity using TAP and KiTAP

Convergent validity was assessed by comparing CAT indices with performance on three subtests of the TAP-M (adults) and KiTAP (children): named Distractibility, Go/NoGo, and Sustained Attention. Raw scores were used for all analyses, as normed scores are currently not available across the full age range. Indeed, no KI-TAP nor TAP-M normed scores were available for the age range of 13 to 18 years old. Hypothesized directions of association are indicated in the result tables using “+” or “-,” and a “not equal” symbol indicates a hypothesized null correlation. Bayesian partial correlations were computed, controlling for age.

Convergent validity in adults was evaluated by correlating CAT indices with TAP-M scores (Table 6A). In the Distractibility subtest, higher TAP-M accuracy during distractor trials was associated with smaller CAT Orienting benefit, whereas greater TAP-M Incorrect Responses are associated with larger CAT Distractor Responses and Anticipated Responses. TAP-M RT variability in no-distractor trials covaried positively with CAT Mean SDRT and negatively with CAT Orienting. In the Go/NoGo task, false alarms were strongly related to CAT Distractor Responses. For Sustained Attention (see Table 7A), TAP-M Correct Responses correlated negatively with CAT Orienting across all time windows. Finally, TAP-M Incorrect Responses showed a positive correlation with CAT Distractor Responses in the 10–15 min block and CAT Arousal in the 0 to 5 min block. No conclusive evidence was found for the other tests.

**Table 6:** Convergent validity: partial Bayesian correlations between CAT indices and KiTAP Distractibility and Go/NoGo subtests in children and adults.

(A)				
TAP-M distractibility	CAT	Expected direction	Results	
			τ	BF10
Correct responses (distractor trials)	Correct responses	+	−0.09	0.495
	Orienting	−	−0.45	9.430 ≠
Correct responses (all trials)	Correct responses	+	0.01	0.449
Correct responses (no distractor trials)	Correct responses	+	0.34	2.163
Incorrect responses (distractor trials)	Distractor responses		0.27	1.219
	Anticipated responses		0.21	0.921
	Cue responses	+	0.11	0.528
	Arousal	+	0.24	0.981
	Distraction	+	0.21	0.854
Incorrect responses (no distractor trials)	Anticipated responses	+	0.37	3.040 ≠
	Cue responses		0.19	0.735
Incorrect responses (all trials)	Distractor responses	+	0.44	8.449 ≠
	Anticipated responses	+	0.34	2.257
	Cue responses		0.19	0.735
	Arousal	+	0.35	2.642
	Distraction	+	0.07	0.473
Missed responses (distractor trials)	Missed Responses (contrast)	−	−0.22	0.886
	Arousal	+	0.24	0.986
Missed responses (all trials)	Missed Responses (contrast)	−	−0.18	0.681
	Arousal	+	0.21	0.879
Missed responses (no distractor trials)	Missed Responses (contrast)	−	0.13	0.557
	Arousal	+	−0.02	0.452
	Late responses (no-distractor)	+	−0.2	0.778
Median RT (distractor trials)	Median RT	+	0.09	0.5
Median RT (all trials)	Median RT	+	0.05	0.465
Median RT (no distractor trials)	Median RT	+	0.01	0.450
RT standard deviation (distractor trials)	Mean SDRT	+	0.23	0.977
RT standard deviation (all trials)	Mean SDRT		0.28	1.277
RT standard deviation (no distractor trials)	Mean SDRT		0.37	3.581 ≠
	Orienting	−	−0.44	8.898 ≠

TAP-M Go-NoGo	CAT	Expected direction	Results	
			τ	BF10
Correct responses	Correct responses	+	0.03	0.467
	Orienting	−	−0.05	0.474
Incorrect responses	Distractor responses	+	0.47	11.791 ≠
	Anticipated responses	+	0.35	2.376
	Cue responses	+	0.13	0.579
	Arousal	+	0.23	0.945
	Distraction		0.16	0.656
Missed responses	Missed Responses (contrast)	−	−0.09	0.501
Median RT	Median RT	+	−0.02	0.459
RT standard deviation	Mean SDRT	+	0.01	0.457

(B)				
KiTAP distractibility	CAT	Expected direction	Results	
			τ	BF10
Correct responses (distractor trials)	Correct responses	+	0.03	0.539
	Orienting	−	−0.09	0.573
Correct responses (all trials)	Correct responses	+	0.00	0.533
Correct responses (no distractor trials)	Correct responses	+	−0.12	0.607
Incorrect responses (distractor trials)	Random responses	+	0.69	85.264 ≠
	Distractor responses		0.15	0.672
	Anticipated responses		0.21	0.839
	Cue responses	+		
	Arousal	+		
	Distraction	+		
Incorrect responses (no distractor trials)	Cue responses	+	0.26	1.070
	Random responses	+	0.76	312.096 ≠
	Anticipated responses	+	0.11	0.605
Incorrect responses (all trials)	Cue responses	+	0.25	1.014
	Random responses	+	0.77	462.385 ≠
	Distractor responses	+	0.14	0.667
	Anticipated responses	+	0.16	0.675
	Arousal	+	0.31	1.470
	Distraction	+	0.09	0.592
Missed responses (distractor trials)	Missed responses (contrast)	−	−0.02	0.534
Missed responses (all trials)	Missed responses (contrast)	−	0.04	0.543
Missed responses (no distractor trials)	Missed responses (contrast)	−	0.18	0.729
	Arousal	+	0.36	2.006
Median RT (distractor trials)	Median RT	+	0.34	1.810
Median RT in (no distractor trials)	Median RT	+	0.51	7.565 ≠
Median RT (all trials)	Median RT	+	0.42	3.517 ≠
RT standard deviation (distractor trials)	Mean SDRT		0.10	0.594
RT standard deviation (no distractor trials)	Mean SDRT		0.07	0.557
RT standard deviation (all trials)	Mean SDRT	+	0.15	0.657

KiTAP Go-NoGo	CAT	Expected direction	Results	
			τ	BF10
Correct responses	Correct responses	+	−0.07	0.544
	Orienting	−	−0.29	1.218
	Distractor responses	+	0.3	1.411
Incorrect responses	Anticipated responses	+	0.09	0.566
	Cue responses	+	0.35	1.936
	Arousal	+	0.31	1.466
	Distraction		−0.15	0.671
Missed responses	Missed responses (contrast)	−	−0.21	0.854
Median RT	Median RT	+	0.53	10.619 ≠
RT standard deviation	Mean SDRT	+	0.4	3.128 ≠

Panel A shows results in adults, and Panel B. shows results in children. All correlations are Kendall’s τ, residualized for age. BF₁₀ values indicate the strength of evidence for an association. “+/−” indicates the a-priori expected direction of the correlation, with no symbol indicating a null hypothesized direction. The ≠ symbol indicates support for the H_1_ hypothesis (correlation), while the = symbolizes support for the H_0_ hypothesis (no correlation).

**Table 7:** Convergent validity: adults’ partial Bayesian correlations between CAT indices and TAP-M sustained-attention subtest.

(A)										
TAP-M sustained attention	CAT	Expected direction	Results							
			0–5 min		5–10 min		10–15 min		All trials	
			τ	BF10	τ	BF10	τ	BF10	τ	BF10
Correct responses (all trials)	Correct responses	+	−0.10	0.526	0.00	0.448	0.06	0.468	−0.01	0.448
	Orienting	−	−0.44	7.972 ≠	−0.34	2.354	−0.39	4.234 ≠	−0.44	8.045 ≠
Incorrect responses (all trials)	Cue responses	+	0.25	1.111	0.17	0.636	−0.10	0.513	0.16	0.645
	Distractor responses	+	0.32	1.875	0.04	0.457	0.48	15.176 ≠	0.41	4.95 ≠
	Arousal	+	0.41	5.563 ≠	0.09	0.501	0.34	2.309	0.42	5.953 ≠
Missed responses (all trials)	Missed responses (contrast)	−	−0.17	0.620	−0.04	0.456	−0.25	1.101	−0.19	0.75
Median RT (all trials)	Median RT	+	0.20	0.767	0.16	0.639	0.26	1.214	0.24	1.003
RT standard deviation (all trials)	Mean SDRT	+	0.08	0.497	−0.02	0.450	0.31	1.807	0.15	0.612

(B)								
KiTAP sustained attention	CAT	Expected direction	Results					
			0–5 min		5–10 min		All trials	
			τ	BF10	τ	BF10	τ	BF10
Correct responses (all trials)	Correct responses	+	−0.33	1.541	0.06	0.533	0.18	0.740
	Orienting	−	0.15	0.665	−0.24	1.018	−0.33	1.717
Incorrect responses (all trials)	Cue responses	+	0.48	6.232 ≠	0.08	0.561	0.15	0.664
	Random responses	+	0.18	0.729	0.03	0.528	0.35	1.826
	Distractor responses	+	0.13	0.623	0.02	0.520	0.13	0.624
	Arousal	+	0.2	0.822	0.11	0.589	0.29	1.273
Missed responses (all trials)	Missed responses (contrast)	−	0.23	0.858	0.33	1.824	0.21	0.853
Median RT (all trials)	Median RT	+	0.46	5.093 ≠	0.54	13.098 ≠	0.6	30.723 ≠
RT standard deviation (all trials)	Mean SDRT	+	0.16	0.687	−0.01	0.520	0.08	0.549

Panel A shows results in adults, and Panel B shows results in children. All correlations are Kendall’s τ, residualized for age. BF₁₀ values indicate the strength of evidence for an association. “+/−” indicates the a-priori expected direction of the correlation, with no symbol indicating a null hypothesized direction. The ≠ symbol indicates support for the H_1_ hypothesis (correlation), while the = symbolizes support for the H_0_ hypothesis (no correlation).

In children, in the Distractibility task, KiTAP Incorrect Responses in both trials with and without a distractor showed very strong positive associations with CAT Random Responses (see Table 6B). In the Go/NoGo task, slower average reaction time and higher intra-individual variability were associated with slower and more variable performance on the CAT, respectively (see Table 6B). For Sustained Attention, KiTAP Median RT correlated with CAT Median RT across all time bins (see Table 7B). KiTAP Incorrect responses during the first 5 min also showed moderate evidence of association with CAT Cue Responses. No conclusive evidence was found for the other tests (0.33 < *BF₁₀* < 3).

Overall, the data show a certain degree of convergence between CAT indices and established TAP-M and KiTAP benchmarks, offering evidence for the CAT convergent validity in childhood and adulthood.

#### 3.2.3 Divergent validity using WAIS and WISC

To assess divergent validity, we examined associations between CAT indices and four subtests from the WISC-V (for children) and WAIS-IV (for adults): Similarities, Matrix Reasoning, Symbol Search, and Coding. These subtests assess domain-general cognitive abilities rather than attention-specific constructs. All analyses used Bayesian correlations. Full results are reported in Supplementary Table 2 (children) and Supplementary Table 3 (adults).

In adults, only Coding displayed a strong negative correlation with Cue Responses, whereas links with Median RT and Arousal were not conclusive. All other CAT-WAIS-IV correlations remained inconclusive (0.33 < *BF₁₀* < 2.99), and none were in favor of H_0_.

In children, the Similarities sub-test score was not found to be associated with any CAT measures. For Matrix Reasoning, two negative correlations exceeded the evidential threshold: Mean SDRT and Late Responses. All other CAT-Matrix pairs had 0.33 < *BF₁₀* < 3, translating to inconclusive results. All other CAT-WISC-V correlations remained inconclusive (0.33 < *BF₁₀* < 2.99), and none were in favor of H_0_.

Together, the negligible correlations with WISC-V and WAIS-IV subtests indicate that the CAT captures attention-specific processes with minimal overlap with domain-general intelligence, thereby supporting both divergent validity and the domain specificity of the CAT across children and adults.

## 4 Discussion

This study provides construct-relevant psychometric evidence for the CAT, demonstrating internal consistency, inter-rater reliability, temporal stability, and theoretically meaningful correlations with external measures. Given the limitations of traditional attention assessments, the CAT fills a gap by measuring both voluntary attentional control and involuntary distractor-driven capture. CAT indices consistently aligned with distinct theoretical constructs, remained robust across examiners, and showed temporal stability. Moreover, clear convergent and divergent validity patterns emerged: CAT indices correlated appropriately with established attention measures while remaining largely independent of broader cognitive skills, such as higher-order reasoning. Together, these findings confirm the CAT’s psychometric quality and its specificity in capturing the behavioral mechanisms underlying distractibility.

### 4.1 Internal consistency

CAT indices organize into theoretically coherent clusters that dissociate voluntary orienting from involuntary capture, arousal, impulsivity, and sustained-attention lapses, supporting the internal consistency and construct specificity of the test. (i) Arousal emerged as a central attentional marker (Aston-Jones and Cohen, 2005; Masson and Bidet-Caulet, 2019), correlating positively with processing speed, reaction time variability, and most notably distractor-driven capture. (ii) Orienting benefit did not correlate substantially with other indices, highlighting that voluntary attentional shifts are psychometrically distinct from the involuntary attention and arousal-driven components (Corbetta and Shulman, 2002; Petersen and Posner, 2012). (iii) Responses to irrelevant events (distractor, cue and anticipated responses) correlated positively among themselves, forming a cluster of impulsivity-related measures. (iv) Missed responses and late responses were negatively correlated, reflecting two mutually exclusive behavioral manifestations of momentary lapses in sustained attention. When attentional engagement transiently drops, participants may either fail to respond altogether (missed responses) or respond with substantial delays (late responses). As these outcomes cannot co-occur within the same trial, individuals who predominantly exhibit one pattern tend to show fewer instances of the other, resulting in a negative association at the interindividual level. In continuous performance and Go/NoGo tasks, increased omission rate (misses) and late responses have been interpreted as markers of lapses of sustained attention (Robertson et al., 1997; O’Connell et al., 2009; Esterman and Rothlein, 2019). In line with this account, reaction time variability (SDRT), a well-established marker of attentional variability, showed convergent associations with both measures, supporting the view that missed and late responses index complementary expressions of the same underlying attentional fluctuations. Altogether, these patterns demonstrate the CAT’s psychometric capacity to differentiate goal-directed voluntary orienting from involuntary capture and sustained attention, while simultaneously capturing the shared variance underlying impulsive or arousal-related errors.

### 4.2 Inter-rater reliability

Complementing internal consistency results, analyses confirmed strong inter-rater reliability, with CAT scores stable across examiners (McHugh, 2012; Shrout and Fleiss, 1979). The few moderate discrepancies observed in reaction-time variability during the initial block likely arose from procedural nuances, such as differences in instruction pacing or participant adjustment to task demands (Streiner et al., 2015). These differences disappeared after the first block, confirming that core CAT indices (speed, accuracy, arousal, and error patterns) remain robust once participants become familiar with the task. Such stability indicates that minimal training and adherence to standardized protocols suffice to ensure reliable administration across research and clinical contexts (Bauer et al., 2012). Nevertheless, future multi-site studies might benefit from brief examiner calibration or digital instruction modules to further reduce early-session variability. Collectively, these findings confirm that the CAT can be administered reliably by different examiners, a robustness essential for multi-site research and clinical scalability.

### 4.3 Test–retest reliability

Over a three-week interval, the CAT demonstrated strong stability for most core indices. An interaction effect between age and session was observed, where young participants tend to have a more pronounced learning curve. This aligns with developmental literature indicating greater sensitivity to learning and repetition due to ongoing maturation of executive control systems (Diamond, 2013; Lin et al., 1999). This result likely reflects small practice-related shifts rather than meaningful changes (McDonald et al., 2002). Indeed, such learning effects are common in attention tests, as participants typically become more proficient at anticipating targets or filtering distractors upon repeated exposure (Calamia et al., 2012). Conversely, older participants showed more stable performance, suggesting that attentional mechanisms become increasingly consistent with age. Although learning-related biases observed here were minimal, future studies could further reduce them through counterbalancing or longer intervals between sessions. Overall, these findings confirm the CAT’s appropriateness for longitudinal monitoring, as most indices exhibit strong short-term reproducibility.

### 4.4 Convergent validity

CAT measures related to omission or commissions, processing speed, and impulsivity displayed the strongest convergent associations with corresponding subtests from the TAP-M and KiTAP. In particular, indices capturing impulsive responses (e.g., anticipated and distractor responses) correlated with error-prone performance on Distractibility and Go/NoGo tasks in both children and adults, highlighting the shared emphasis on inhibitory control (Catale et al., 2009; Verbruggen and Logan, 2008; Zimmerman and Fimm, 2002). Processing speed indices, such as median reaction time, also converged reliably with similar reaction time measures on these batteries. Meanwhile, attention orientation effects in the CAT correlated with tasks that require spatially directed attention (Posner, 1980). For instance, while the CAT explicitly provides directional cues for voluntary orienting, the TAP-M and KiTAP often embed orienting demands within a broader context of executive control (e.g., interleaving distractors or mixed task instructions). Consequently, although the tasks share core features of attention and inhibitory control, the magnitude of correlations varies depending on the degree to which each subtest isolates, or conflates, involuntary capture, voluntary orienting, and higher-order executive processes. Importantly, these convergent associations underscore that the CAT captures fundamental aspects of attentional functioning in a manner consistent with established clinical tools, while at the same time offering complementary perspectives that may enhance the ecological and developmental sensitivity of attentional assessment.

### 4.5 Divergent validity

CAT indices generally did not correlate with Similarities or Matrix Reasoning, two subtests designed to measure higher-order verbal and abstract reasoning, respectively, rather than attentional processes (Sudarshan and Bowden, 2023; Wechsler, 2008, 2014). While attention is typically a cognitive requirement for the realisation of these subtests, the absence of association between CAT and Wechsler’s measures supports the idea that distractibility-related behaviors in the CAT are not simply derivatives of fluid intelligence or verbal skills (Engle et al., 1999; Joy et al., 2004). By contrast, we observed a moderate association between certain CAT measures and Coding or Symbol Search, both of which tap processing speed and quick visual scanning (Joy et al., 2004; Mayes and Calhoun, 2006). These associations appear to reflect the shared component of rapid information processing rather than distractibility itself (Salthouse, 2010). Overall, the CAT’s specificity, showing minimal overlap with more complex cognitive abilities, reinforces its utility as a targeted tool for measuring attention and distractibility.

### 4.6 Developmental trajectories across experiments

A recurring pattern in the present study is how age or developmental status modulates the reliability and validity of the CAT indices. Although both children and adults exhibit strong internal consistency for impulsivity-related measures (e.g., anticipated and distractor responses), the clustering of these indices appears more robust in adults, suggesting that impulsive behaviors may consolidate into a stable profile after adolescence (Hoyer et al., 2021; Rubia, 2013). Children, in contrast, exhibited increased variability on certain measures, particularly in test–retest comparisons, where younger participants showed more pronounced fluctuations in anticipated responses and reaction time variability. These age-by-session interactions align with prior evidence that executive functions and motor inhibition networks continue to mature well into late adolescence (Diamond, 2013), making them more susceptible to practice effects or day-to-day fluctuations (Luna et al., 2015; Ordaz et al., 2013). From a validity standpoint, although children and adults both demonstrated convergent associations with the TAP-M or KiTAP and divergent associations with Wechsler subtests, children sometimes showed weaker or inconsistent correlations for certain CAT indices with these external measures. One plausible explanation is that developing cognitive capacities may be less specialized, with overlapping processes (e.g., arousal, impulsivity, working memory) influencing a wide range of tasks (Casey et al., 2008; Diamond, 2013; Hoyer et al., 2021; Thillay et al., 2015). Adults, by contrast, appear to differentiate attentional processes more distinctly, leading to stronger or more targeted correlations between CAT measures and external tasks. These age differences are in line with established neurodevelopmental trajectories in frontoparietal and ventral networks, which progressively refine top-down/voluntary control and bottom-up/involuntary capture over adolescence (Casey et al., 2008; Diamond, 2013; Luna et al., 2015; Petersen and Posner, 2012; Thillay et al., 2015). Furthermore, known aging effects in older adulthood (e.g., increased susceptibility to distraction) might parallel shifts observed in the CAT’s arousal-related indices, underlining that arousal changes during the lifespan (ElShafei et al., 2020a; Hoyer et al., 2023a; Lau et al., 2001; Yoon et al., 1998). Overall, the CAT’s capacity to highlight developmental changes in attention and other related abilities highlights its utility in research and potential clinical contexts that require a nuanced assessment of how distractibility and impulse behavior evolve with age. By capturing both stable and modifiable aspects of attention, the CAT offers a lens into the interplay between neurodevelopmental changes in cortical circuits and the behavioral manifestations of distractibility.

### 4.7 Clinical and neuroscientific implications

The CAT’s psychometric properties support its relevance for cognitive and developmental research. By disentangling involuntary capture from impulsive false alarms, the CAT could further conceptually help clinicians refine their understanding of attentional profiles, where anticipatory errors often prevail (Barkley, 1997; Conners, 2014; Catale et al., 2009; McDonald et al., 2002). However, the present study does not address clinical sensitivity or diagnostic utility. While the CAT demonstrates sound psychometric properties, data from clinical populations, such as individuals with ADHD, are required to evaluate its potential diagnostic value and to establish sensitivity and specificity for routine clinical use. Beyond diagnostic profiling, the CAT’s strong test–retest reliability suggests its potential utility in monitoring intervention outcomes that target attentional dysfunction. Improvements on specific indices of the CAT (e.g., fewer impulsive responses, reduced distractor-driven capture or reaction time variability) could serve as tangible endpoints in pharmacological trials, psycho-educative interventions, or neuromodulation experiments. Additionally, its design, capturing both top-down and bottom-up components of attention (Bidet-Caulet et al., 2015), offers valuable foundations for EEG or fMRI investigations seeking to map attentional networks in real-time (ElShafei et al., 2018, 2020b). Indeed, by simulating ecologically valid distractions, the CAT bridges the gap between controlled laboratory tasks and everyday attention demands, facilitating research into how cortical circuits manage distraction under realistic conditions.

### 4.8 Limitations

Nevertheless, certain limitations remain. For instance, our cohort was entirely French-speaking and neurotypical, limiting cross-cultural or clinical inferences. The three-week retest interval is also relatively modest: further studies are required to test the long-term stability of the CAT measures. Minor ceiling effects in adult accuracy could reduce variability in reaction times in this population. Inter-rater reliability analyses were restricted to adolescents and adults due to practical constraints in experimenter overlap for younger participants, and therefore conclusions from these analyses should not be generalized to the youngest age groups. On another note, using a single age covariate across heterogeneous samples assumes a uniform relationship between age and attentional measures, an assumption that may not fully capture stage-specific or non-linear developmental effects across the lifespan. Finally, we did not conduct factor analysis, expert content mapping, or tests of measurement invariance: as such, this study does not provide structural validation. The findings instead reflect construct-relevant evidence based on observed-score coherence, test–retest stability, and theoretically guided associations. Importantly, the absence of clinical populations restricts our ability to draw robust conclusions regarding the CAT diagnostic sensitivity and specificity: evaluating these properties in clinical samples therefore would constitutes a logical and necessary extension of the present work.

## 5 Conclusion

This study provides robust psychometric evidence for the CAT as a measure of distractibility, using ecologically valid stimuli to capture distinct attentional processes. Although formal sensitivity and specificity remain to be established, the CAT reliably indexes both attentional voluntary and involuntary influences, showing strong internal consistency, structured relationships among speed, arousal, and impulsivity indices, acceptable test–retest stability, and clear patterns of convergent and divergent validity. While most indices demonstrated robust reliability and validity, a few, particularly those related to false alarms or arousal shifts, showed only modest stability and may be more sensitive to task repetition or age-related variability. Together, these properties support the CAT’s utility for cognitive neuroscience research and indicate its potential for future clinical application, pending further validation of its diagnostic accuracy.

## Data Availability

The datasets presented in this article are not readily available because authors RH and AB-C developed an application reproducing the test used in this study (trademarked as “CoLeT”). This application relies on a normative database built from the data reported here; therefore, the raw data cannot be shared. Requests to access the datasets should be directed to Roxane.Hoyer@cervo.ulaval.ca.
