# Supplementary material for: Validation of the competitive attention test: behavioral reliability and construct-relevant associations across the lifespan
Source: Front Psychol. 2026 May 21;17:1720923. doi: 10.3389/fpsyg.2026.1720923 (PMC13233474; doi:10.3389/fpsyg.2026.1720923)
Supplement: Supplementary file 1 [file Table_1.docx]

**Supplementary table 1**

*Standardized effect sizes quantifying the age-adjusted Session-related mean shift (Cohen’s d)*

| **Measure** | **Coefficient b** | **σ** | **Cohen's d** |
| --- | --- | --- | --- |
| **median_RT** | -25.71 | 41.06 | -0.63 |
| **mean_SDRT** | -25.64 | 63.10 | -0.41 |
| **Orienting** | 4.57 | 36.3 | 0.13 |
| **Arousal** | 19.61 | 40.59 | 0.48 |
| **Capture** | -0.78 | 39.38 | -0.02 |
| **CorRep** | -1.93 | 8.60 | -0.23 |
| **CueRep** | -0.14 | 0.44 | -0.32 |
| **RandRep** | -0.04 | 0.41 | -0.11 |
| **DisRep** | -0.02 | 2.92 | -0.01 |
| **AntRep** | 1.88 | 2.70 | 0.70 |
| **LateRep_NoDis** | -0.35 | 4.47 | -0.08 |
| **LateRep_DisNoDis** | -0.98 | 4.45 | -0.22 |
| **MissRep_NoDis** | 0.87 | 7.18 | 0.12 |
| **MissRep_DisNoDis** | 0.43 | 3.43 | 0.13 |

*Note*. Coefficient b represents the age-adjusted fixed effect of Session on each measure. σ corresponds to the residual standard deviation. Cohen’s d quantifies the standardized mean shift between sessions, computed as b/σ. Negative values indicate lower scores at the second session relative to the first, whereas positive values indicate higher scores.

## Supplementary Table 2

***Divergent validity: Children’s*** *Bayesian correlations between CAT indices and WISC‑V subtests.*

| **WISC-V** | **CAT** | **τ** | **BF10** |
| --- | --- | --- | --- |
| Similarities | Median RT | 0.09 | 0.573 |
|  | Orienting | 0.15 | 0.655 |
|  | Arousal | 0.05 | 0.544 |
|  | Correct Responses | -0.14 | 0.682 |
|  | Cue Responses | -0.20 | 0.794 |
|  | Distractor Responses | -0.07 | 0.567 |
|  | Anticipated Responses | -0.18 | 0.727 |
|  | Late Responses (no-distractor) | -0.15 | 0.654 |
| Matrix | Median RT | 0.26 | 1.038 |
|  | Mean SDRT | -0.49 | 6.271 |
|  | Orienting | 0.02 | 0.533 |
|  | Arousal | -0.19 | 0.757 |
|  | Correct Responses | -0.04 | 0.541 |
|  | Cue Responses | -0.12 | 0.622 |
|  | Distractor Responses | -0.19 | 0.744 |
|  | Anticipated Responses | 0.17 | 0.711 |
|  | Late Responses (no-distractor) | -0.67 | 55.267 ≠ |
| Symbol Search | Median RT | -0.08 | 0.571 |
|  | Mean SDRT | -0.10 | 0.589 |
|  | Orienting | -0.09 | 0.578 |
|  | Arousal | -0.28 | 1.181 |
|  | Correct Responses | 0.06 | 0.552 |
|  | Cue Responses | -0.15 | 0.677 |
|  | Distractor Responses | -0.21 | 0.819 |
|  | Anticipated Responses | -0.04 | 0.541 |
|  | Late Responses (no-distractor) | -0.20 | 0.754 |
| Coding | Median RT | -0.33 | 1.590 |
|  | Mean SDRT | -0.02 | 0.533 |
|  | Orienting | 0.07 | 0.560 |
|  | Arousal | -0.27 | 1.083 |
|  | Correct Responses | 0.21 | 0.846 |
|  | Cue Responses | 0.00 | 0.532 |
|  | Distractor Responses | 0.12 | 0.628 |
|  | Anticipated Responses | 0.09 | 0.577 |
|  | Late Responses (no-distractor) | 0.06 | 0.548 |

*Note.* All correlations are Kendall’s τ, residualized for age. BF₁₀ values indicate the strength of evidence for an association. “+/-” indicates the a‑priori expected direction of the correlation, with no symbol indicating null hypothesized direction. The ≠ symbol indicates support for H_1_ hypothesis, while the = symbolizes support for the H_0_ hypothesis.

## **Supplementary** Table 3

***Divergent validity: Adults’*** *Bayesian correlations between CAT indices and WAIS‑IV subtests.*

| **WAIS-IV** | **CAT** | **τ** | **BF10** |
| --- | --- | --- | --- |
| Similarities | Median RT | 0.15 | 0.624 |
|  | Orienting | -0.10 | 0.520 |
|  | Arousal | 0.06 | 0.479 |
|  | Correct Responses | 0.02 | 0.450 |
|  | Cue Responses | -0.14 | 0.584 |
|  | Distractor Responses | -0.07 | 0.479 |
|  | Anticipated Responses | -0.02 | 0.450 |
|  | Late Responses (no-distractor) | -0.02 | 0.455 |
| Matrix | Median RT | 0.09 | 0.509 |
|  | Mean SDRT | 0.04 | 0.460 |
|  | Orienting | -0.12 | 0.547 |
|  | Arousal | 0.09 | 0.498 |
|  | Correct Responses | -0.04 | 0.462 |
|  | Cue Responses | 0.13 | 0.594 |
|  | Distractor Responses | 0.05 | 0.465 |
|  | Anticipated Responses | 0.03 | 0.453 |
|  | Late Responses (no-distractor) | -0.13 | 0.577 |
| Symbol Search | Median RT | 0.20 | 0.816 |
|  | Mean SDRT | -0.01 | 0.449 |
|  | Orienting | -0.24 | 1.003 |
|  | Arousal | 0.16 | 0.661 |
|  | Correct Responses | -0.02 | 0.452 |
|  | Cue Responses | -0.16 | 0.641 |
|  | Distractor Responses | 0.14 | 0.597 |
|  | Anticipated Responses | 0.05 | 0.468 |
|  | Late Responses (no-distractor) | -0.29 | 1.460 |
| Coding | Median RT | 0.29 | 1.576 |
|  | Mean SDRT | -0.03 | 0.452 |
|  | Orienting | -0.06 | 0.478 |
|  | Arousal | 0.32 | 1.799 |
|  | Correct Responses | 0.15 | 0.611 |
|  | Cue Responses | -0.49 | 19.507 ≠ |
|  | Distractor Responses | -0.08 | 0.495 |
|  | Anticipated Responses | -0.06 | 0.475 |
|  | Late Responses (no-distractor) | -0.28 | 1.317 |

*Note.* All correlations are Kendall’s τ, residualized for age. BF₁₀ values indicate the strength of evidence for an association. “+/-” indicates the a‑priori expected direction of the correlation, with no symbol indicating null hypothesized direction. The ≠ symbol indicates support for H_1_ hypothesis, while the = symbolizes support for the H_0_ hypothesis.
